# Therapeutic modalities in the first cancer treatment: percentage variation before and during the pandemic, in Ceará, a state in the Northeast region of Brazil

**DOI:** 10.1590/1980-549720250024

**Published:** 2025-05-09

**Authors:** Stefane Vieira Nobre, Miren Maite Uribe Arregi, Thereza Maria Magalhães Moreira, Antonio Germane Alves Pinto, Marcelo Gurgel Carlos da Silva

**Affiliations:** I Universidade Estadual do Ceará – Fortaleza (CE), Brazil.; II Centro Regional Integrado de Oncologia – Fortaleza (CE), Brazil.; III Universidade Regional do Cariri – Fortaleza (CE), Brazil.

**Keywords:** Neoplasms, Epidemics, COVID-19, Therapeutics, Epidemiology

## Abstract

**Objective:**

To evaluate the percentage variation of the different therapeutic modalities used in new cases of cancer, before and during the pandemic, in Ceará.

**Methods:**

Descriptive study with information from records of the Hospital Information System and Authorization of Highly Complex Procedures, and Cancer Information Systems in the state of Ceará, Brazil.

**Results:**

There was a reduction of 2,575 (-30.85%) new cases in 2020 and 2,368 (-28.37%) in 2021. Regarding the therapeutic modalities, it was possible to observe that there were decreases in the numbers of all institutions in the first cancer treatment, except for isolated radiotherapy and for combined surgery+iodine therapy+radiotherapy. Surgery was the modality most often performed in a timely manner (<30 days) and chemotherapy in an untimely manner (>60 days). The impact on the initiation of timely treatment (<30 days after diagnosis) was greater in 2021 than in 2020. In general, late treatments (>60 days) were lower than in 2019. The period of 31-60 days was shown to be above 2019 almost the entire year, in 2020 and 2021.

**Conclusion:**

The number of new cancer cases in Ceará may occur during the pandemic. This situation impacted the reduction of all therapeutic modalities, except for radiotherapy alone and associated with surgery+iodine therapy. However, it did not harm the number of new cases treated in a timely manner.

## INTRODUCTION

Cancer is the second leading cause of death worldwide, claiming over 9.7 million lives in 2022^1^. In developing countries, incidence and mortality rates continue to rise or remain stable compared to those in developed nations^2^. In Brazil, cancer accounted for 232,040 deaths in 2019, with 51,874 occurring in the Northeast region^3^. According to estimates from the National Cancer Institute (*Instituto Nacional de Câncer* – INCA) for 2023, Ceará recorded approximately 31,390 new cancer cases^2^.

Currently, cancer intervention and care involve three primary treatment modalities: surgery, chemotherapy, and radiotherapy. These approaches can be used in combination, with variations and adaptations based on the tumor's susceptibility to each method and the sequence of administration. Notably, few malignant neoplasms are treated with a single modality^4^.

Investigating the direct and indirect impacts of COVID-19 enables the assessment of both current and future implications while also informing cancer care policies in the context of pandemics with significant societal effects, such as COVID-19. Beyond its substantial impact on cancer patient survival, the limitations imposed by the pandemic may lead to delayed diagnoses, ultimately increasing the burden on the healthcare system with higher costs compared to detecting the disease in its preclinical phase^5,6^.

Conducting the research in Ceará is justified for practical reasons, as the state provides oncology care to its 184 municipalities through two High Complexity Oncology Assistance Centers (*Centros de Assistência de Alta Complexidade em Oncologia* – Cacon) and seven High Complexity Assistance Units (*Unidades de Assistência de Alta Complexidade* – Unacon)^7^.

In light of these considerations, the following question emerged: "Has the COVID-19 pandemic impacted the treatment of new cancer cases?" The study hypothesizes that, due to the risk of COVID-19 contamination, treatment protocols were modified to prioritize patient safety. The objective was to assess the percentage variation in the use of different therapeutic modalities for new cancer cases in Ceará before and during the pandemic.

## METHODS

This descriptive study was conducted using data on outpatient and hospital cancer treatments from oncology reference units and centers in the state of Ceará^7^.

The data were collected from the Ceará State Health Secretariat (*Secretaria de Saúde do Estado do Ceará* – Sesa/CE) and sourced from records in the Hospital Information System (*Sistema de Informações Hospitalares* – SIH), the Authorization for High Complexity Procedures (*Autorização de Procedimentos de Alta Complexidade* – Apac), and the Oncology Panel of the SUS Information Technology Department (*Departamento de Informática do SUS* – DataSUS)^8^.

Data collection was conducted in May and June 2022. The study included data on cancer treatment provided through the Brazilian Unified Health System (*Sistema Único de Saúde* – SUS) at oncology reference centers and units in Ceará. The pre-pandemic reference period was 2019, compared to the period between 2020 and 2021, when COVID-19 was prevalent. Although the pandemic began in Brazil in March 2020, data from January and February 2020 were also included to assess potential differences in monthly case numbers before the pandemic.

The following inclusion criteria were adopted for data collection at Sesa/CE: patient data related to outpatient and hospitalization procedures involved in the care of cancer patients, covering all types of cancer. Exclusion criteria included: Apac records with zero values or incomplete data; incomplete Apac and Hospital Admission Authorization (*Autorização de Internação Hospitalar* – AIH) forms; and treatments that were initiated in previous years.

For data collection in the Oncology Panel, the following variables were considered: Federative Unit of residence (Ceará); year of diagnosis (2019–2021); treatment duration (<30, 31–60, >60 days); Diagnosis [Malignant Neoplasms (Law No. 12.732/12)], and month/year of treatment (January/2019 to December/2021). For the analysis of treatment initiation time according to therapeutic modality, the same variables were used, along with the additional variable of therapeutic modality (surgery, chemotherapy, radiotherapy, and their combinations). Cases lacking treatment information were excluded.

The information was extracted from Apac and AIH records, organized by the years comprising the historical series (2019, 2020, and 2021), and entered into an Excel spreadsheet version 2019^®^ (Microsoft Corporation, Redmont, Washington DC, USA). Since the database included all procedures performed between 2019 and 2021, a key variable was created to remove duplicates by combining the National Health Card (*Cartão Nacional de Saúde* – CNS) number with the diagnosis, as per the International Classification of Diseases (ICD). After eliminating duplicates, only the first treatment performed was considered. Outpatient treatments not corresponding to the study years within the series were also filtered and excluded.

For the descriptive analysis of treatment quantity and type, frequency measures (absolute and relative) and percentage variation were used.

The Term of Faithful Depositary with Sesa/CE was used to authorize the collection of documents. The research was approved by the Ethics and Research Committee of Universidade Estadual do Ceará (CEP/UECE) under opinion number 5.353.022, dated 04/15/2022.

## RESULTS

The total number of cancer cases diagnosed in 2019 was used as a reference, as the pandemic began at the end of that year, and no cases were reported in Brazil in 2019. In 2020, there was a reduction of 2,575 (-30.85%) in new patients with neoplasms initiating treatment. Monthly reductions exceeding this annual decline were observed in January (-43.10%), May (-54.90%), June (-46.79%), and July (-43.17%). In 2021, the number of newly treated cases decreased by 2,368 (-28.37%). The most significant declines occurred in January (-48.33%), February (-43.90%), and May (-41.74%), as shown in Table 1.

**Table 1:** Monthly and annual percentage variation of new cases of malignant neoplasms treatment in Ceará, 2019–2021. Ceará, Brazil, 2022.

Month	2019	2020	2021	PV*	PV†
	n	n	n	%	%
January	956	544	494	-43.10	-48.33
February	934	565	524	-39.51	-43.90
March	657	560	507	-14.76	-22.83
April	653	510	445	-21.90	-31.85
May	745	336	434	-54.90	-41.74
June	639	340	555	-46.79	-13.15
July	695	395	492	-43.17	-29.21
August	641	489	470	-23.71	-26.68
September	591	502	503	-15.06	-14.89
October	662	517	504	-21.90	-23.87
November	613	467	511	-23.82	-16.64
December	561	547	540	-2.50	-3.74
Total	8,347	5,772	5,979	-30.85	-28.37

Sources: Hospital Information System, Authorization for High Complexity Procedures, Oncology Panel of the SUS Informatics Department, Individualized Outpatient Production Bulletin, and Cancer Information System.

PV: percentage variation.

* monthly and annual percentage variation from 2019 to 2020;

† monthly and annual percentage variation from 2019 to 2021.

In Table 2, the therapeutic modalities were detailed, revealing declines in the number of all treatment modalities used for initial cancer treatment, except for isolated radiotherapy and the combined approach of surgery+iodine therapy+radiotherapy.

**Table 2:** Number of new cancer treatments by therapeutic modality in the state of Ceará, Brazil, during the COVID-19 pandemic (2020 and 2021), compared to the year immediately preceding the pandemic (2019).

Treatment Adopted	2019	2020	2021	PV*	PV†
	n	n	n	%	%
Surgery	4,067	2,781	3,067	-31.62	-24.59
Chemotherapy	1,903	938	913	-50.71	-52.02
Radiotherapy	987	1,040	1,162	5.37	17.73
Iodotherapy	23	5	8	-78.26	-65.22
Surgery+Iodotherapy	16	11	12	-25.41	-25.00
Surgery+Chemotherapy	307	229	174	-25.41	-43.32
Surgery+Radiotherapy	95	88	52	-7.37	-45.26
Surgery+Chemotherapy+Radiotherapy	237	210	121	-11.39	-48.95
Surgery+Iodotherapy+Radiotherapy	0	1	0	0.00	0.00
Chemotherapy+Radiotherapy	712	469	470	-34.13	-33.99
Total	8,347	5,772	5,979	-31.62	-24.59

Sources: Hospital Information System, Authorization for High Complexity Procedures, Oncology Panel of the SUS Informatics Department, Individualized Outpatient Production Bulletin, and Cancer Information System.

PV: Percentage variation.

* annual percentage variation between 2019 and 2020;

† annual percentage variation between 2019 and 2021.

Regarding the distribution of time delay categories for treatment initiation by therapeutic modality, surgery was the most frequently performed within the timely period (<30 days), while chemotherapy was most often initiated late (>60 days). A decline was observed across all modalities in the <30-day period, with the most significant reduction in surgery. In the 31-to-60-day range, a decrease was noted only for radiotherapy. Beyond 60 days, radiotherapy showed the greatest reduction. In 2021, surgery continued to experience the largest reduction, while chemotherapy was the only modality to increase. Within the 31-to-60-day period, radiotherapy exhibited the greatest decline, whereas chemotherapy and combined modalities increased. In the >60-day category, all modalities declined, with chemotherapy showing the most pronounced reduction. However, an increase in chemotherapy initiation within <30 days was observed, as shown in Table 3.

**Table 3:** Distribution of time intervals (in days) between cancer diagnosis and the start of treatment, by treatment modality, from 2019 to 2021. Ceará, Brazil, 2022.

Modality	2019			2020			2021			Total
	<30	31–60	>60	<30	31–60	>60	<30	31–60	>60	
Surgery	4,572	79	305	3,805	83	219	3,483	61	168	12,775
Chemotherapy	1,303	1,342	2,519	1,592	1,437	2,222	1,666	1,713	2,277	16,071
Radiotherapy	517	660	1,766	493	630	1,385	260	545	1,195	7,451
Both	20	18	28	19	21	31	9	19	31	196
Total	6,412	2,099	4,618	5,909	2,171	3,826	5,418	2,338	3,671	36,462

Source: SUS Informatics Department. Oncology Panel. 2022.

Note: Both=chemotherapy+radiotherapy with the same treatment date.

**Figure 1:**
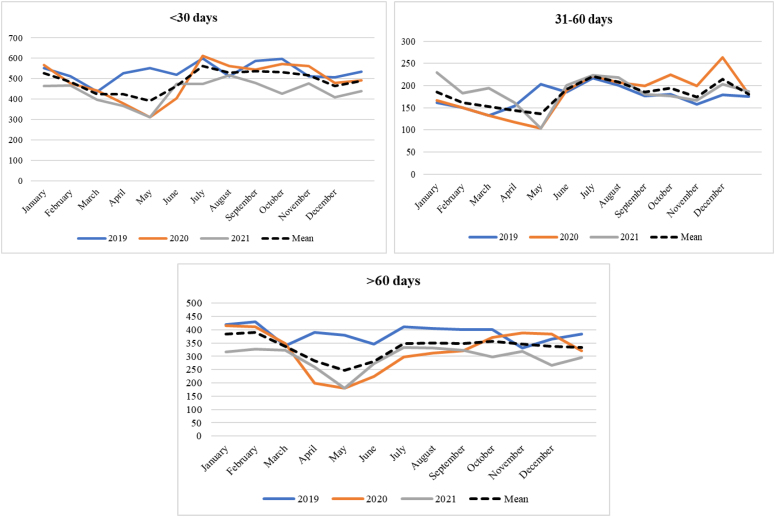
Distribution of time intervals (in days) between cancer diagnosis and the start of treatment in Ceará, 2019–2021.

Source: SUS Informatics Department. Oncology Panel. 2022.

The monthly and annual distributions of initial treatment for new cancer cases, categorized by the time between diagnosis and treatment initiation (<30 days, 31–60 days, >60 days), are presented in Figure 1. In 2020, the most significant declines across all time periods occurred in May. However, a recovery was observed after May for treatments initiated within <30 days; in the 31-to-60-day period, subsequent months showed declines exceeding those of 2019; while in the >60-day category, declines persisted until the last two months of the year. In 2021, the most notable declines were recorded in May, July, and October, with overall treatment numbers remaining lower than in 2019 throughout the year. In the 31-to-60-day period, a decline was observed in May, but treatment numbers subsequently aligned with 2019 levels. In the >60-day category, a decrease was also observed in May, with treatment numbers remaining below those of 2019 in the following months.

## DISCUSSION

A reduction in new neoplasm cases initiating treatment was observed in January, May, June, and July 2020, as well as in January, February, and May 2021, compared to the year immediately preceding the pandemic.

One study reported that between March and July 2020, there were reductions of 37,667 and 59,340 in clinical and surgical hospitalizations related to cancer, respectively, compared to the same period in 2019^9^. Another study identified a 21% decline in hospitalizations in 2020 relative to the estimated value based on the average of previous years, along with a 14% increase in the mortality rate among hospitalized cancer patients^10^. Additionally, a referral center observed a 43% reduction in total hospitalizations (from 13,791 to 7,860) and a 59.3% decrease in surgeries performed (from 12,621 to 5,133) during the pandemic compared to the pre-pandemic period^11^.

The disruptions in cancer treatments stem from patients’ fear of exposure to SARS-CoV-2, leading them to avoid seeking specialized care. Additional contributing factors include the implementation of social isolation measures to control SARS-CoV-2 transmission, reduced availability of public transportation, postponement of initial consultations, cancellation of elective and screening procedures, potential interruptions in the drug supply chain, reassignment of oncology specialists to frontline COVID-19 care, and the reallocation of resources to support the treatment of infected patients^10,12^.

In 2020, the most significant declines in treatments for new cancer cases occurred in January, May, June, and July, marking the first phase of the pandemic. This period was characterized by the spread of cases to the interior regions^13^. One of the initial measures implemented was social isolation, which restricted transportation between different areas of the country. This restriction may have contributed to the decline in treatments for new cancer cases at Unacon and Cacon, as these facilities are located in hub municipalities, some of which are more remote, making intermunicipal travel more challenging. The study "ConVid – Behavior Research" (*ConVid - Pesquisa de Comportamentos*) identified that the presence of Noncommunicable Diseases (NCDs) was associated with greater adherence to social distancing, increased demand for healthcare, and difficulty in accessing health services^14^.

In 2021, reductions in cancer treatments occurred in January, February, and May, corresponding to the second and third waves of the pandemic. During the second wave, transmission and death rates stabilized, but the number of cases increased, along with high test positivity, and the number of hospitalizations and deaths remained steady. The third wave was characterized as the second and deadliest wave of the COVID-19 pandemic across the country. This period saw a widespread crisis in the healthcare system, with peaks of up to 4,000 deaths per day and a 7-day moving average exceeding 3,100 deaths. It is important to note that, during this time, the lack of care not only contributed to higher mortality from COVID-19 but also to increased deaths from untreated chronic conditions^13^.

Although the phases of the pandemic severely impacted the Brazilian population as a whole, it is important to note that there was heterogeneous behavior across the country's states, with some states showing a more rapid rise in cases and deaths. Siqueira et al.^15^ assessed trends in COVID-19 incidence and mortality across Brazil's federal units and capitals and found that Ceará experienced the fastest reduction in cases during the second wave (a monthly reduction of 52.4%). Among the capitals, Fortaleza had the fastest reduction in cases during the second wave, following Palmas (42.9% reduction), Rio Branco (47.1% reduction), and Belém (46.9% reduction). The greatest reduction in deaths occurred in Fortaleza during the first wave, with a decrease of 51.8%.

The declines in the number of new cancer treatments observed during the two years of the study were primarily concentrated in chemotherapy, radiotherapy, and chemotherapy+radiotherapy combinations. A study conducted with a Brazilian supplementary health agency, which analyzed administrative data from a private health insurance company covering 41,640 people over two 90-day periods (one before and one during the pandemic), found reductions of 14.1% in chemotherapy, 28.1% in radiotherapy, 35% in exams, and 49.4% in hospital admissions^16^. However, results from a different study involving users of another private health insurance plan indicated that chemotherapy and radiotherapy did not experience significant declines^17^.

The use of surgery to remove superficial soft tissue masses, particularly those less than 5 cm in diameter, is a common practice in non-specialized centers, assuming the mass is benign. After the material is collected, a histopathological diagnosis is promptly made and recorded in the Oncology Panel within 30 days. This process may help explain the predominance of the surgical modality in the category of timely cancer treatment^18-20^

Chemotherapy and radiotherapy, whether administered alone or in combination, are often not performed in a timely manner due to the need for referrals to specialized reference centers. These centers typically face high demand for outpatient procedures, leading to delays in the initiation of therapy^7,21^.

The impact on timely treatment was more significant in 2021 than in 2020. Overall, the late initiation of treatments for new cancer cases decreased compared to 2019. This trend may be attributed to the lower number of appointments during the pandemic, which may have allowed for better management of treatment demand. A study analyzing delays in the initiation of oral cancer treatment in Brazil between 2013 and 2019 found that the goal of early treatment initiation was achieved in 61% of cases nationwide in 2019. The study also highlighted that the years with the highest frequency of treatment within the recommended period were 2018 and 2019, with a noted increase in the percentage of cases starting treatment within 30 days in the Northeast region of the country^22^.

It is important to note that Federal Law No. 12.732/2012 guarantees the right of cancer patients to have their treatment initiated within 60 days, establishing this timeframe as a legal requirement for the start of treatment^23^.

Regarding the distribution of time between diagnosis and treatment according to the treatment modality, surgery was the most frequently performed modality within the timely manner (<30 days), while chemotherapy was the most frequently delayed modality (>60 days). These findings are consistent with the literature and the perspectives discussed above, which highlight that outpatient treatments (chemotherapy and radiotherapy) experienced more significant reductions during the study period. Surgical treatments, in contrast, are typically executed more quickly than outpatient treatments.

This study provides a short-term historical overview of the effects of COVID-19 on cancer therapy in the state of Ceará. Long-term repercussions, however, can be better assessed in the coming years as the impacts of pent-up demand throughout the phases and peaks of COVID-19 are measured. In 2020, University College London projected that at least 6,270 additional deaths could occur in England over the next 12 months among people with new cancer diagnoses, representing a 20% increase. If all individuals with cancer were considered, this number could rise to 17,915 deaths. The reduction in chemotherapy and consultations during the pandemic is cited as the primary cause of this increase in mortality^24^.

The results of this research allowed for the identification, clarification, and quantification of the repercussions arising from the COVID-19 pandemic on new cancer cases eligible for outpatient and surgical treatments. An overview of the number of treatments, the most affected modalities, and the period between diagnosis and the start of treatment was provided to support actions and policies by health managers. A key distinction of this study is its focus on the state of Ceará, where few studies have addressed this issue. Although the COVID-19 pandemic impacted all states in the country, the repercussions in terms of cases and deaths were felt heterogeneously across regions.

The main limitation of this study is the incompleteness of some data recorded in the AIH and APAC, such as the date of diagnosis and the start of treatment, as well as the lack of detailed information about the modality or medication listed in outpatient treatments. Another limitation is the significant amount of treatment data not recorded in the Oncology Panel, which prevents a comprehensive analysis of the entire population undergoing treatment. Some variables could not be examined due to missing information in the oncology case surveillance systems.

The repercussions measured in this study focused on possible short-term changes, as data from the first two years of the pandemic were analyzed. It is important to emphasize the need for studies covering longer periods, as well as prospective longitudinal studies that can monitor the long-term repercussions of the pandemic on cancer treatment and outcomes.

There were reductions in treatments for new cancer cases during the COVID-19 pandemic; however, these reductions occurred during specific periods in each pandemic year studied. All therapeutic modalities experienced a reduction, except for radiotherapy when used alone or in combination with surgery+iodine therapy. Additionally, there was a decrease in the timely initiation of treatment. However, a decrease was also observed in the late initiation of treatment (>60 days), with an increase in the initiation of treatment within the period between 31–60 days after diagnosis.
