# Supplementary material for: Therapeutic modalities in the first cancer treatment: percentage variation before and during the pandemic, in Ceará, a state in the Northeast region of Brazil
Source: Rev Bras Epidemiol. 2025 May 9;28:e250024. doi: 10.1590/1980-549720250024 (PMC12068809; doi:10.1590/1980-549720250024)
Supplement: Translation

# Translation: Modalidades terapêuticas no primeiro tratamento do câncer: variação percentual antes e durante a pandemia, no Ceará, um estado da região Nordeste do Brasil

**DOI:** 10.1590/1980-549720250024.2

**Authors:** Stefane Vieira Nobre, Miren Maite Uribe Arregi, Thereza Maria Magalhães Moreira, Antonio Germane Alves Pinto, Marcelo Gurgel Carlos da Silva

**Affiliations:** I Universidade Estadual do Ceará – Fortaleza (CE), Brasil.; II Centro Regional Integrado de Oncologia – Fortaleza (CE), Brasil.; III Universidade Regional do Cariri – Fortaleza (CE), Brasil.

**Keywords:** Neoplasias, Epidemia, COVID-19, Terapêutica, Epidemiologia

## Abstract

**Objetivo:**

Avaliar a variação percentual das diferentes modalidades terapêuticas empregadas nos casos novos de câncer, antes e durante a pandemia, no Ceará.

**Métodos:**

Estudo descritivo com informações de registros do Sistema de Informações Hospitalares, da Autorização de Procedimentos de Alta Complexidade e dos Sistemas de Informação do Câncer no Estado do Ceará, Brasil.

**Resultados:**

Houve redução de 2.575 (-30,85%) casos novos em 2020 e 2.368 (-28,37%) em 2021. Quanto às modalidades terapêuticas, pôde-se observar que houve quedas nos números de todas instituídas no primeiro tratamento de câncer, exceto para radioterapia isolada e para cirurgia+iodoterapia+radioterapia combinadas. A cirurgia foi a modalidade mais realizada em tempo oportuno (<30 dias) e a quimioterapia em tempo não oportuno (>60 dias). O impacto no início do tratamento oportuno (<30 dias do diagnóstico) foi maior em 2021 que em 2020. Em geral, os tratamentos tardios (>60 dias) foram menores que em 2019. O período de 31–60 dias mostrou-se quase o ano todo acima de 2019, em 2020 e 2021.

**Conclusão:**

O número de casos novos por câncer no Ceará reduziu durante a pandemia. Essa situação impactou na redução de todas as modalidades terapêuticas, exceto radioterapia isolada e associada à cirurgia+iodoterapia. Entretanto, não reduziu o número de casos novos tratados em tempo oportuno.

## INTRODUÇÃO

O câncer ocupa o segundo lugar em causas de mortes no mundo e em 2022 ceifou a vida de mais de 9,7 milhões de pessoas^1^. Nos países em desenvolvimento, as taxas de incidência e mortalidade seguem aumentando ou estáveis quando comparadas aos países desenvolvidos^2^. No Brasil, em 2019 houve 232.040 mortes por câncer, sendo 51.874 na região Nordeste^3^. De acordo com a estimativa do Instituto Nacional de Câncer (INCA) para 2023, o Ceará atingiu um número de 31.390 casos estimados de câncer^2^.

Atualmente as linhas de intervenção e cuidado ao câncer dispõem de três principais modalidades de tratamento, a saber: cirurgia, quimioterapia e radioterapia. Estas podem ser usadas de forma combinada, podendo tais combinações serem variadas e adaptadas conforme à suscetibilidade tumoral a cada um dos métodos citados e à sequência de administração. Ressalta-se que poucas neoplasias malignas são tratadas com apenas uma modalidade^4^.

Desse modo, investigar as mudanças e os impactos ocasionados pela COVID-19, direta e indiretamente, permite avaliar as implicações atuais e futuras desses danos, além de subsidiar ações para políticas assistenciais ao câncer em condições de pandemias com grande impacto na sociedade, como foi a de COVID-19. Além das repercussões significativas na sobrevida dos pacientes com câncer, as limitações impostas pela pandemia poderão causar diagnósticos tardios e, consequentemente, onerar gastos maiores ao sistema de saúde em comparação à descoberta da doença na fase pré-clínica^5,6^.

Por oportuno, a realização da pesquisa no Ceará justifica-se por ser um estado que fornece atendimento aos seus 184 municípios através de 2 Centros de Assistência de Alta Complexidade em Oncologia (Cacon) e 7 Unidades de Assistência de Alta Complexidade (Unacon)^7^.

Destarte ao exposto, emergiu o seguinte questionamento: "A pandemia de COVID-19 impactou no tratamento de novos casos de câncer?". A hipótese apresentada pelo estudo é que, em virtude do risco de contaminação pela COVID-19, os tratamentos sofreram alterações em seu curso para preservar a integridade do paciente. O estudo teve como objetivo avaliar a variação percentual das diferentes modalidades terapêuticas empregadas nos casos novos de câncer, antes e durante a pandemia, no Ceará.

## MÉTODOS

Estudo descritivo realizado a partir de informações sobre tratamentos ambulatoriais e hospitalares de câncer em unidades e centros de referência em cuidado ao câncer no estado do Ceará^7^.

As informações foram coletadas na Secretaria de Saúde do Estado do Ceará (Sesa/CE), oriundas dos registros do Sistema de Informações Hospitalares (SIH), da Autorização de Procedimentos de Alta Complexidade (Apac) e do Painel Oncologia do Departamento de Informática do SUS (DataSUS)^8^.

O período de coleta dos dados compreendeu maio e junho de 2022. Integraram ao estudo dados de tratamento de pacientes com câncer, por meio do Sistema Único de Saúde (SUS), em centros e unidades de referência em tratamento de câncer no Ceará. Em 2019, teve-se a referência comparativa pré-pandemia em relação ao período entre 2020 e 2021, de ocorrência da COVID-19. Embora a pandemia tenha iniciado no Brasil a partir de março de 2020, a pesquisa considerou os meses de janeiro e fevereiro de 2020, com a finalidade de verificar se houve diferença nos casos mensais antes da pandemia.

Para a coleta de dados na Sesa/CE foram adotados como critérios de inclusão: dados de pacientes em relação a procedimentos ambulatoriais e internação que estivessem envolvidos no cuidado a pacientes com câncer, incluindo dados sobre todos os tipos de câncer. Foram excluídos: Apac zeradas e não concluídas; Apac e Autorização de Internação Hospitalar (AIH) sem preenchimento completo e tratamentos iniciados em outros anos.

Para a coleta no Painel Oncologia: Unidade Federativa (UF) de residência (Ceará); ano do diagnóstico (2019–2021); tempo de tratamento (<30, 31–60, >60 dias); Diagnóstico [Neoplasias Malignas (Lei n° 12.732/12)], mês/ano de tratamento (janeiro/2019 a dezembro/2021). Para os dados sobre tempo de início de tratamento segundo modalidade terapêutica foram utilizadas as mesmas variáveis, acrescidas da variável modalidade terapêutica (cirurgia, quimioterapia, radioterapia, e suas combinações). Foram excluídos casos sem informação de tratamento.

As informações foram extraídas das Apac e AIH, separadas pelos anos que compuseram a série histórica (2019, 2020 e 2021) e colocadas em planilha Excel versão 2019^®^ (Microsoft Corporation, Redmont, Washington DC, USA). Uma vez que o banco registrava todos os procedimentos realizados entre 2019 e 2021, foi criada uma variável chave para retirada de duplicidades através da junção do número do Cartão Nacional de Saúde (CNS) com o diagnóstico estabelecido de acordo com a Classificação Internacional de Doenças (CID). Retiradas as duplicidades, foi considerado somente o primeiro tratamento realizado. Também foram filtrados e excluídos os tratamentos ambulatoriais que não correspondiam aos anos estudados dentro da série.

Para análise descritiva dos valores referentes à quantidade e modalidade de tratamentos foram empregadas medidas de frequência (absoluta e relativa) e variação percentual.

Utilizou-se o termo de fiel depositário junto à Sesa/CE para autorização da coleta dos documentos. A pesquisa obteve aprovação através do Comitê de Ética e Pesquisa da Universidade Estadual do Ceará (CEP/Uece), com o número de parecer 5.353.022, datado de 15/04/2022.

## RESULTADOS

Tomou-se como referência o total de casos de câncer diagnosticados no ano 2019, uma vez que o início da pandemia foi no final deste ano e que não houve relato de casos no Brasil em 2019. Assim, no ano 2020 houve redução de 2.575 (-30,85%) novos pacientes com neoplasias a iniciarem o tratamento. Nos meses, a redução acima deste valor anual ocorreu em: janeiro (-43,10%), maio (-54,90%), junho (-46,79%) e julho (-43,17%). Em 2021, houve diminuição de 2.368 (-28,37%) casos iniciais a serem tratados. Foram observadas quedas em janeiro (-48,33%), fevereiro (-43,90%) e maio (-41,74), conforme observado na Tabela 1.

**Tabela 1:** Variação percentual mensal e anual de tratamentos de casos novos de neoplasias malignas no Ceará, 2019–2021. Ceará, Brasil, 2022.

Mês	2019	2020	2021	VP*	VP†
	n	n	n	%	%
Janeiro	956	544	494	-43,10	-48,33
Fevereiro	934	565	524	-39,51	-43,90
Março	657	560	507	-14,76	-22,83
Abril	653	510	445	-21,90	-31,85
Maio	745	336	434	-54,90	-41,74
Junho	639	340	555	-46,79	-13,15
Julho	695	395	492	-43,17	-29,21
Agosto	641	489	470	-23,71	-26,68
Setembro	591	502	503	-15,06	-14,89
Outubro	662	517	504	-21,90	-23,87
Novembro	613	467	511	-23,82	-16,64
Dezembro	561	547	540	-2,50	-3,74
Total	8.347	5.772	5.979	-30,85	-28,37

Fontes: Sistema de Informações Hospitalares, Autorização de Procedimentos de Alta Complexidade, Painel Oncologia do Departamento de Informática do SUS, Boletim de Produção Ambulatorial Individualizado e Sistema de Informações de Câncer.

VP: variação percentual.

* variação percentual mensal e anual de 2019 e 2020;

† variação percentual mensal e anual de 2019 e 2021.

Na Tabela 2 foram discriminadas as modalidades terapêuticas, onde pôde-se observar que houve quedas nos números de todas as modalidades terapêuticas instituídas no primeiro tratamento de câncer, exceto para radioterapia isolada e para cirurgia+iodoterapia+radioterapia combinadas.

**Tabela 2:** Número de tratamentos de casos novos de câncer segundo a modalidade terapêutica no estado do Ceará, Brasil, durante a pandemia de covid-19 (2020 e 2021), em comparação com o ano imediatamente anterior à pandemia (2019).

Tratamento adotado	2019	2020	2021	VP*	VP†
	n	n	n	%	%
Cirurgia	4067	2781	3067	-31,62	-24,59
Quimioterapia	1903	938	913	-50,71	-52,02
Radioterapia	987	1040	1162	5,37	17,73
Iodoterapia	23	5	8	-78,26	-65,22
Cirurgia+iodoterapia	16	11	12	-25,41	-25,00
Cirurgia+quimioterapia	307	229	174	-25,41	-43,32
Cirurgia+radioterapia	95	88	52	-7,37	-45,26
Cirurgia+quimioterapia+radioterapia	237	210	121	-11,39	-48,95
Cirurgia+iodoterapia+radioterapia	0	1	0	0,00	0,00
Quimioterapia+radioterapia	712	469	470	-34,13	-33,99
Total	8.347	5.772	5.979	-31,62	-24,59

Fontes: Sistema de Informações Hospitalares, Autorização de Procedimentos de Alta Complexidade, Painel Oncologia do Departamento de Informática do SUS, Boletim de Produção Ambulatorial Individualizado e Sistema de Informações de Câncer.

VP: variação percentual.

* variação percentual anual entre 2019 e 2020;

† variação percentual anual entre 2019 e 2021.

Em se tratando da distribuição das categorias do tempo de demora para início do tratamento, de acordo com a modalidade terapêutica empregada, observou-se que a cirurgia consistiu na modalidade mais realizada em tempo oportuno (<30 dias) e que a quimioterapia foi a modalidade mais desempenhada em um tempo não oportuno (>60 dias). Houve redução em todas as modalidades no período <30 dias, com maior redução na cirurgia. O período 31–60 dias apresentou decréscimo somente na radioterapia. Acima de 60 dias foi observado maior decréscimo na radioterapia. No ano de 2021 a cirurgia seguiu sendo a mais reduzida, havendo acréscimo somente na quimioterapia. Entre 31–60 dias obteve-se maior decréscimo na radioterapia, havendo aumento na quimioterapia e em ambos. No período acima de 60 dias houve redução em todas as modalidades, mas predominou a redução da quimioterapia; houve aumento de tratamento desta modalidade no período <30 dias, conforme Tabela 3.

**Tabela 3:** Distribuição dos intervalos de tempo (em dias) entre diagnóstico e início de tratamento de câncer, segundo modalidade de tratamento, no período 2019–2021. Ceará, Brasil, 2022.

Modalidade	2019			2020			2021			Total
	<30	31–60	>60	<30	31–60	>60	<30	31–60	>60	
Cirurgia	4.572	79	305	3.805	83	219	3.483	61	168	12.775
Quimioterapia	1.303	1.342	2.519	1.592	1.437	2.222	1.666	1.713	2.277	16.071
Radioterapia	517	660	1.766	493	630	1.385	260	545	1.195	7.451
Ambos	20	18	28	19	21	31	9	19	31	196
Total	6.412	2.099	4.618	5.909	2.171	3.826	5.418	2.338	3.671	36.462

Fonte: Departamento de Informática do SUS. Painel-Oncologia. 2022.

Nota: Ambos=quimioterapia+radioterapia com a mesma data de tratamento.

As distribuições mensal e anual de tratamento inicial de casos novos de câncer, segundo períodos entre o diagnóstico e o início do tratamento (<30 dias, 31–60 dias, >60 dias) estão descritas na Figura 1. Em 2020 foram observadas quedas mais expressivas no mês de maio em todos os períodos supracitados. Entretanto, houve recuperação das quedas após o mês de maio no período <30 dias; entre 31–60 dias os meses seguintes superaram as quedas em comparação a 2019; e >60 dias a superação das quedas ocorreu somente nos dois últimos meses. Em 2021, as quedas foram mais expressivas em maio, julho e outubro, sendo o ano todo com números inferiores a 2019. Entre 31–60 dias, houve queda em maio, mas na sequência os números acompanharam os achados de 2019. No período >60 dias também houve queda em maio, e nos meses seguintes os valores se mostraram abaixo daqueles em 2019.

**Figura 1:** Distribuição dos intervalos de tempo (em dias) entre diagnóstico e início de tratamento de câncer no Ceará, 2019–2021.

Fonte: Departamento de Informática do SUS. Painel-Oncologia. 2022.

## DISCUSSÃO

Houve redução de casos novos de neoplasias a iniciarem tratamento nos meses de janeiro, maio, junho e julho de 2020; e em janeiro, fevereiro e maio de 2021, quando comparados ao ano imediatamente pré-pandemia.

Um estudo observou que no período de março a julho de 2020 houve reduções de 37.667 e 59.340 internações clínicas e cirúrgicas relacionadas ao câncer, respectivamente, quando comparadas ao mesmo período de 2019^9^. Outro estudo também identificou queda de 21% nas internações em 2020, comparado ao valor estimado para o ano baseado na média dos anos anteriores; ainda, a taxa de mortalidade de pacientes hospitalizados com câncer aumentou 14%^10^. Um centro de referência também observou redução de 43% (de 13.791 para 7.860) no número total de internações, e de 59,3% (de 12.621 para 5.133) nas cirurgias realizadas no período da pandemia, quando comparados ao período pré-pandêmico^11^.

As repercussões ocorridas nos tratamentos do câncer vêm do receio do paciente em ser exposto ao SARS-CoV-2, consequentemente evitando a procura aos serviços especializados. Outras causas envolvem a implementação do isolamento social como medida de controle da infecção por SARS-CoV-2, redução do transporte público, adiamento de consultas de primeira vez, cancelamento de procedimentos eletivos e de rastreio, risco de interrupção da cadeia de fornecimento de medicamentos, desvio de profissionais de saúde especializados em oncologia para trabalhar na linha de frente, e recursos redirecionados para apoiar o atendimento de pacientes infectados^10,12^.

Em 2020, as maiores quedas nos tratamentos para casos novos de câncer ocorreram em janeiro, maio, junho e julho, compreendendo a primeira fase. Esse período foi caracterizado pela interiorização dos casos^13^. Uma das primeiras medidas adotadas foi o isolamento social, que restringiu o transporte entre diversas localidades do país. Esse período pode ter colaborado para a redução dos tratamentos de casos novos de câncer nas Unacon e Cacon, uma vez que funcionam em municípios-polo, sendo alguns mais distantes, o que torna o translado intermunicipal mais difícil. O estudo "ConVid - Pesquisa de Comportamentos" identificou que a presenta de Doenças e Agravos Não Transmissíveis (DANT) esteve associada à maior adesão de distanciamento social, procura por atendimento de saúde e dificuldade no acesso aos serviços de saúde^14^.

Em 2021, a redução se deu nos meses de janeiro, fevereiro e maio, compreendendo a segunda e a terceira onda. Na segunda onda foi observada uma estabilização nos indicadores de transmissão e óbitos e elevação no número de casos, alta positividade de testes e estabilização do número de internações e óbitos. A terceira fase foi caracterizada como a segunda e mais mortal onda da pandemia de COVID-19 em todo o país. Instaurou-se uma crise generalizada do sistema de saúde, com picos de até 4 mil óbitos por dia e média móvel de 7 dias superior a 3.100 óbitos. Salienta-se que nesse período a desassistência não só oportunizou uma maior mortalidade por COVID-19, como por diversas doenças crônicas não assistidas^13^.

Embora as fases da pandemia tenham assolado severamente a população brasileira em sua totalidade, é válido destacar que houve um comportamento heterogêneo nos estados do país, de forma que alguns estados se sobressaíram mais rapidamente em relação à incidência de casos e óbitos. Siqueira et al.^15^ avaliaram as tendências de incidência e de mortalidade por COVID-19 nas unidades de federação e capitais do Brasil e observou que o Ceará apresentou a maior velocidade de redução de casos na segunda onda (redução mensal de 52,4%). Já entre as capitais, Fortaleza apresentou maior velocidade de redução de casos na segunda onda, ficando atrás de Palmas (redução de 42,9%), Rio Branco (redução de 47,1%) e Belém (redução de 46,9%). A maior redução de óbitos ocorreu em Fortaleza, na primeira onda (redução de 51,8%).

As quedas observadas na quantidade de novos tratamentos para casos de câncer se concentraram nas quimioterapias, iodoterapias e na combinação quimioterapia+radioterapia nos dois anos estudados, respectivamente. Em estudo desenvolvido com uma agência de saúde suplementar brasileira coletaram-se, em 41.640 pessoas, dados administrativos de uma operadora privada de saúde relativos a dois períodos de 90 dias, um antes e outro durante a pandemia. Observou-se redução de 14,1% nas quimioterapias, 28,1% nas radioterapias, 35% nos exames realizados e 49,4% nas internações hospitalares^16^. Entretanto, os resultados de um estudo desenvolvido com usuários de outro plano privado de saúde trouxeram dados distintos quando mostraram que quimioterapias e radioterapias não sofreram queda expressiva^17^.

O uso da cirurgia para retirada de massas superficiais de tecidos moles, principalmente de tamanho inferior a 5 cm de diâmetro, é uma prática comum nos centros não especializados, mediante a presunção de que a massa não possua teor maligno. Logo após a coleta do material é feito um diagnóstico histopatológico com posterior registro no Painel-Oncologia dentro do intervalo de até 30 dias. Depreende-se que esse trâmite pode explicar a predominância da modalidade cirúrgica na categoria de tempo oportuno de tratamento para o câncer^18-20^

Já as modalidades quimioterapia e radioterapia, quando aplicadas de forma isolada ou em associação, tendem a não ser realizadas em tempo oportuno, uma vez que dependem de encaminhamentos para centros de referência, que usualmente apresentam uma grande demanda para desempenho de procedimentos ambulatoriais, resultando em um atraso no início da terapêutica^7,21^.

O impacto no tratamento oportuno foi maior em 2021 que em 2020. Em geral, os inícios tardios de tratamentos para casos novos de câncer tiveram redução em relação a 2019. Tal achado poderia ser decorrente da menor quantidade de atendimentos durante a pandemia e melhor possibilidade de atendimento à demanda de tratamentos. Ao analisar o tempo de demora para o início do tratamento do câncer de boca no Brasil, no período entre 2013 e 2019, observou-se que o objetivo para início precoce do tratamento foi alcançado em 61% dos casos no país em 2019. Ainda de acordo com o estudo, os anos com maior frequência de tratamento no período preconizado foram 2018 e 2019, além da análise temporal ter identificado uma variação percentual crescente no período <30 dias para início do tratamento na região Nordeste do país^22^.

Ressalta-se que o estabelecimento do tempo de até 60 dias para início do tratamento ao paciente oncológico é garantido por meio da Lei Federal n° 12.732/2012, que define como direito do paciente com câncer ter seu tratamento iniciado no prazo em questão^23^.

Quanto à distribuição de tempo entre diagnóstico e tratamento segundo a modalidade de tratamento, a cirurgia consistiu na modalidade mais realizada em tempo oportuno (<30 dias), enquanto a quimioterapia foi a modalidade mais desempenhada em um tempo não oportuno (>60 dias). Os achados corroboram a literatura e as perspectivas discutidas acima em relação às modalidades ambulatoriais (quimioterapia e radioterapia) terem sofrido maiores reduções no período estudado. Há uma maior celeridade na execução do tratamento cirúrgico em detrimento do ambulatorial.

Este estudo traz um panorama histórico de curto prazo acerca dos efeitos da COVID-19 na terapêutica oncológica no estado do Ceará, haja vista que as repercussões a longo prazo poderão ser melhor avaliadas no curso dos próximos anos após serem mensurados os impactos nas demandas reprimidas ao longo das fases e dos picos da COVID-19. Em 2020, verificou-se, na University College London, que pelo menos 6.270 mortes adicionais poderiam acontecer na Inglaterra nos 12 meses seguintes em pessoas com novos diagnósticos de câncer, ou seja, um incremento de 20%. Caso todas as pessoas com câncer fossem consideradas, esse número poderia subir para 17.915 mortes. Atribui-se a causa à redução de quimioterapias e consultas durante a pandemia^24^.

Os resultados desta pesquisa permitiram identificar, elucidar e quantificar a presença de repercussões oriundas da pandemia de COVID-19 nos casos novos de câncer que foram elegíveis a tratamentos ambulatoriais e cirúrgicos. Foi traçado um panorama do número de tratamentos, as modalidades mais afetadas e o período entre diagnóstico e início de tratamento, com vistas a fornecer informações para subsidiar ações e políticas pelos gestores em saúde. O estudo também traz um diferencial mediante sua abordagem ter sido feita no estado do Ceará, onde ainda há poucos estudos que abordem essa questão. Embora a pandemia de COVID-19 tenha afetado todos estados do país, as repercussões dos casos e óbitos foram sentidas de forma heterogênea entre eles.

A fragilidade principal do estudo consiste na incompletude de algumas informações preenchidas nas AIH e Apac, como data de diagnóstico e início de tratamento, e a falta de detalhamento acerca da modalidade ou do medicamento elencado no tratamento ambulatorial. Também se evidencia como fragilidade a grande quantidade de informações sobre tratamentos não registradas no Painel-Oncologia, o que inviabiliza uma análise de toda a população em tratamento, uma vez que algumas variáveis não podem ser estudadas devido à deficiência de informações nos sistemas de vigilância dos casos oncológicos.

As repercussões aqui mensuradas analisaram possíveis alterações a curto prazo, pois foram avaliados os dados dos dois primeiros anos de pandemia. Salienta-se a necessidade de serem elaborados estudos que possam abranger maiores períodos, além de estudos longitudinais prospectivos que acompanhem as repercussões a longo prazo.

Houve reduções nos tratamentos de casos novos de câncer durante a pandemia de COVID-19, entretanto, aconteceram em uma parcela de tempo em cada ano pandêmico estudado. Houve redução de todas as modalidades terapêuticas, exceto radioterapia isolada e associada à cirurgia+iodoterapia. Também observou-se uma diminuição do início oportuno do tratamento, entretanto também houve queda no início tardio do tratamento (>60 dias), uma vez que foi identificado aumento no início de tratamento no período entre 31–60 dias do diagnóstico.
